# Acute effects of the RAMP warm-up on sprint and jump performance in youth soccer players

**DOI:** 10.3389/fphys.2025.1612611

**Published:** 2025-06-24

**Authors:** Fatma Gözlükaya Girginer, Sinan Seyhan, Görkem Açar, Muhammed Fatih Bilici, Ömer Faruk Bilici, Çağlar Soylu

**Affiliations:** ^1^ Department of Coaching Education, Faculty of Sport Sciences, Pamukkale University, Denizli, Türkiye; ^2^ Department of Coaching Education, Faculty of Sport Sciences, Manisa Celal Bayar University, Manisa, Türkiye; ^3^ Department of Sport Science, Institute of Graduate Education, Manisa Celal Bayar University, Manisa, Türkiye; ^4^ Muş Alparslan Üniversitesi, Spor Bilimleri Fakültesi, Antrenörlük Eğitimi Bölümü, Muş, Türkiye; ^5^ Department of Movement and Training, Marmara University, Istanbul, Türkiye; ^6^ Gulhane Faculty of Physiotherapy and Rehabilitation, University of Health Sciences, Ankara, Türkiye

**Keywords:** warm-up, ramp protocol, static stretching, neuromuscular activation, vertical jump, sprint performance, pre-competition, soccer

## Abstract

**Introduction:**

Pre-competition warm-ups play a critical role in optimizing athletic performance and minimizing injury risk. This randomized, controlled crossover study investigated the acute effects of the Raise, Activate, Mobilize, and Potentiate (RAMP) warm-up protocol on key performance parameters in young male soccer players.

**Methods:**

Fourteen participants (aged 16–22 years) completed three different warm-up conditions—no warm-up (control), static stretching, and RAMP—on non-consecutive days with a 48-h washout period. Performance was assessed using 30-m sprint and vertical jump tests, each performed twice per session with the best trial recorded for analysis.

**Results:**

A one-way repeated-measures analysis of variance (ANOVA) revealed that both vertical jump height and sprint performance differed significantly across conditions (p < 0.05). Post hoc Bonferroni comparisons indicated that the RAMP group exhibited superior results compared with static stretching (Effect size: d = 0.41) and control (Effect size: d = 0.52), while no notable difference was observed between static stretching and control conditions.

**Discussion:**

These results suggest that the structured progression of the RAMP protocol—combining exercises that elevate body temperature, activate key muscle groups, improve mobility, and enhance neuromuscular readiness—can acutely enhance explosive strength and sprint speed by effectively priming the neuromuscular system. This study contributes novel insights by demonstrating the acute efficacy of the RAMP method in youth soccer players, a population that has been underrepresented in previous literature. Although the study was limited to male amateur athletes and focused on short-term performance gains, the findings indicate suggestive potential for implementing the RAMP protocol as an evidence-based approach in pre-competition preparation.

## 1 Introduction

Pre-competition warm-up is a well-established practice in athletic preparation, widely recognized for its role in enhancing athletic performance and reducing injury risk (Ramos-Campo et al., 2020). By preparing the muscular and nervous systems for the physical demands of competition, effective warm-up routines improve movement efficiency and neuromuscular coordination while mitigating the likelihood of acute musculoskeletal injuries (Afonso et al., 2023). In recent years, there has been a growing emphasis on the scientific validation of warm-up strategies, highlighting the need for evidence-based protocols tailored to specific athletic populations (McGowan et al., 2015; McMillian et al., 2006). Although several studies have examined long-term adaptations, substantial literature also investigates the acute effects of various warm-up strategies across different populations and contexts (Fradkin et al., 2010; Curry et al., 2009). Recent literature emphasizes the importance of individualized, progressive warm-up strategies to improve performance and reduce injury risk. For example, Washif et al. (2025) outline current best practices in warm-up structure, noting that most sports programs now include dynamic and sport-specific components. While numerous protocols exist, including static stretching, dynamic movements, and combined methods, consensus on the most effective approach remains limited due to variability in sport-specific demands and study designs.

A wide array of warm-up modalities—including aerobic exercises, dynamic and static stretching—has been investigated in the context of athletic performance (Samson et al., 2012; Bishop, 2003; Curry et al., 2009; Faigenbaum et al., 2005). While dynamic warm-ups are generally associated with performance enhancement due to their ability to increase muscle temperature, neural drive, and stretch-shortening cycle efficiency, static stretching has been shown to transiently impair power, speed, and strength outputs—likely due to reduced musculotendinous stiffness and neural inhibition—without significantly reducing injury risk (Herda et al., 2008; Samson et al., 2012; Jeffreys, 2019). Dynamic stretching has gained attention due to its ability to acutely enhance performance metrics by increasing muscle temperature, enhancing neural drive, and improving movement efficiency.

Emerging frameworks highlight that incorporating personalized and autonomy-supportive elements into warm-up routines may improve both psychological engagement and adherence, especially in youth athletes. Such approaches were believed to increase intrinsic motivation and may contribute to improved performance outcomes by fostering routine consistency and athlete buy-in (Fradkin et al., 2010; Huéscar et al., 2020; Silva et al., 2018). Recent literature has also explored complementary approaches—such as foam rolling and explosive dynamic drills—that may enhance neuromuscular readiness by improving blood flow, joint range of motion, and motor unit recruitment, particularly in both elite and youth athletes (Popelka et al., 2024; Skopal et al., 2024). However, their comparative effectiveness across different sports and age groups remains underexplored. Nevertheless, studies evaluating the transferability and efficacy of these protocols across different sports and developmental stages remain scarce.

The RAMP framework aligns with modern warm-up paradigms that emphasize progressive activation, mobility, and neuromuscular readiness (Barbosa et al., 2025a). The RAMP protocol consists of four sequential phases: (1) Raise–to increase body temperature and heart rate through low-intensity aerobic activity; (2) Activate–to engage key muscle groups using dynamic drills like lunges and leg swings; (3) Mobilize–to improve joint range of motion via mobility-focused tasks such as hurdle steps and lateral shuffles; and (4) Potentiate–to prepare for high-intensity efforts through explosive movements such as maximal sprints (Jeffreys, 2019). Previous studies have applied RAMP-based warm-ups in contexts such as change-of-direction training for police students (Kukić et al., 2024) and volleyball-specific protocols for youth athletes (Cieśluk et al., 2024), demonstrating improvements in agility and reaction time, respectively. However, more data are needed regarding its immediate efficacy in soccer performance.

Dynamic stretching, known to enhance power output and reduce injury risk, is a key component of contemporary warm-up protocols and merits further discussion when comparing RAMP to other methods. Therefore, the present study aims to investigate the acute effects of the RAMP warm-up protocol on key performance parameters—including vertical jump height, sprint speed, and agility—in young soccer players. By assessing these motor abilities immediately following the RAMP intervention, this study seeks to address a gap in the literature regarding evidence-based warm-up protocols tailored for youth athletes. The findings are anticipated to contribute to the optimization of pre-competition preparation in soccer and similar high-intensity sports. It is hypothesized that the RAMP protocol will result in significant short-term improvements in lower-limb explosive power, sprint performance, and agility compared to a traditional warm-up routine. This hypothesis builds upon prior findings suggesting that multi-component warm-up protocols can acutely improve explosive movements in youth athletes (Faigenbaum et al., 2005; Kukić et al., 2024).

## 2 Materials and methods

### 2.1 Participants and study design

This randomized, controlled crossover study included 16 male amateur soccer players aged between 16 and 22 years, all of whom had at least 2 years of structured football training and participated in regular weekly sessions (1–3 h/week). Goalkeepers and individuals with any current musculoskeletal injuries were excluded to ensure a homogenous sample and reliable measurement of motor performance variables. The final sample consisted of 14 participants with a mean age of 21.86 ± 0.86 years, mean height of 180.42 ± 2.59 cm, mean body mass of 73.43 ± 7.90 kg, and a mean BMI of 22.07 ± 2.76 kg/m^2^. According to self-reported positions, the sample included six midfielders, four defenders, and 4 forwards. The study protocol was approved by the Ethics Committee of Pamukkale University (Approval No: E-60116787-020-666811, Date: 04.02.2025), and was conducted in accordance with the Declaration of Helsinki. Participants and/or their legal guardians were informed in detail about the aims, procedures, and possible risks of the study, and provided written informed consent prior to participation. A crossover experimental design was employed, with participants randomly assigned via the Randomize.org platform to undergo three distinct warm-up protocols: (i) no warm-up (control condition, CG), (ii) static stretching protocol (SSG), and (iii) the RAMP warm-up protocol (RPG). Each participant completed all three protocols on non-consecutive days (48-h washout), with the order of testing counterbalanced to mitigate order effects. All sessions were conducted between 09:00 and 11:00 to avoid diurnal variations. To determine the minimum required sample size, a power analysis was conducted using G*Power software. Based on a one-way repeated-measures ANOVA design and using a conventional effect size as recommended in previous literature (Kang, 2021), with an alpha level of 0.05 and power of 0.80, the required sample size was estimated to be 14 participants. Thus, 16 participants were recruited to account for potential dropouts.

### 2.2 Experimental procedure

The study was conducted at Pamukkale University, where participants trained four times per week and competed in official matches once per week. All testing sessions were carried out between 09:00 and 11:00 a.m. in a rested state to control for circadian variability. Participants were instructed to abstain from intense physical activity, caffeine, tea, alcohol, carbonated beverages, and other stimulants for at least 24 h prior to each testing session.

The experimental timeline spanned three non-consecutive days.

• Day 1 (Baseline): Anthropometric measurements (height and body mass) were taken. This was followed by the administration of the 30-m sprint test and the vertical jump test without any prior warm-up.
• Day 2: The static stretching protocol (SSG) was applied, after which the same two performance tests were repeated (Table 1).
• Day 3: The RAMP warm-up protocol (RPG) was implemented, and performance assessments were conducted once more (Table 2).

**TABLE 1:** Static stretching protocol (SSG).

Exercise	Description
Quadriceps stretch	Heel pulled to the buttocks in standing position
Standing hamstring stretch	Trunk flexion toward the toes while standing
Seated wide-leg stretch	Forward reach in long sitting position
Seated toe touch	Trunk flexion in long sitting, arms reaching toward toes
Standing calf stretch	Against wall or static object

Note: Each exercise was performed bilaterally, held for ∼30 s, and conducted through a pain-free range of motion.

**TABLE 2:** Detailed structure of the RAMP warm-up protocol applied in this study.

Phase	Duration	Exercises
Raise	∼3 min	- Light jogging - Straight-leg high knees - High knees (right/left) - Lateral gallop - Carioca (crossover step)
Activate	∼10 min	- Arm circles (forward/backward) - Dynamic high knees (bilateral) - Forward lunges - Butt kicks (right/left) - Backward running - Leg swings (inward/outward)
Mobilize	∼5 min	- High-knee over-hurdle (inward/outward) - Crossover steps over mini-hurdles (right/left) - Lateral shuffle over hurdle (right/left) - Straight-leg hurdle kicks (right/left)
Potentiate	∼2 min	−2 × 30 m maximal sprints - 1-min rest between repetitions

Although participants were exposed to all three warm-up conditions in a counterbalanced order with washout periods, random allocation to sequence groups was not fully achieved. Therefore, the design is best described as a quasi-randomized crossover. All performance tests were repeated twice per session, and participants were given 2 minutes of passive rest between attempts. The best performance value from each test was used for final analysis. To ensure reliability of the repeated trials, the test-retest reliability of both the vertical jump and sprint tests was assessed. The intraclass correlation coefficient (ICC) for the 30-m sprint test was 0.91 (CV = 2.1%), and for the vertical jump test using the My Jump two app was 0.93 (CV = 1.8%), indicating excellent reliability across sessions.

#### 2.2.1 RAMP protocol (RPG)

The RAMP protocol included four sequential phases, designed to physiologically and neurologically prepare athletes for high-intensity activity (Table 2). All exercises were performed at moderate to high intensity, progressively increasing neuromuscular activation. Each component was designed to address a specific physiological function, including thermoregulation, muscle activation, dynamic flexibility, and potentiation of power output (Jeffreys, 2019).

### 2.3 Performance tests and data collection instruments

While only two performance tests were selected (vertical jump and 30-m sprint), the decision was based on their strong validity as proxies for lower-limb explosive strength and sprint performance in soccer players. The inclusion of additional assessments (e.g., agility T-test, change-of-direction drills) was considered but excluded to avoid participant fatigue and maintain standardization across repeated sessions. Future studies may expand on this by including more complex motor tasks.

#### 2.3.1 Anthropometric measurements

Participants’ height (cm) and body mass (kg) were measured using a calibrated stadiometer and a digital weighing scale, respectively. The scale had a sensitivity of ±0.01 kg, and the height was recorded to the nearest ±0.1 cm. During measurement, participants stood barefoot, with their heels together, back straight, and head in the Frankfurt plane.

#### 2.3.2 30-Meter sprint test

Participants began from a standing start 50 cm behind the initial gate, which allowed them to establish a natural acceleration phase before reaching the first timing sensor. This distance was selected based on prior protocols used in similar studies (Santander et al., 2022), and was deemed sufficient to initiate movement without prematurely triggering the photocell system. The sprint performance distance was 30 m.

#### 2.3.3 Vertical jump test

Explosive power was assessed using the My Jump two application, a validated tool that calculates vertical jump height from high-speed video recordings (Silva et al., 2024; Gür and Ayan, 2023). The following procedure was applied.

• A 240 fps video was recorded using an iPhone 11 placed 1 m away from the lateral aspect of the participant.
• Participants performed a bilateral countermovement jump with hands on hips to eliminate arm swing effects.
• The app automatically identified take-off and landing frames based on foot-ground contact.
• Variables recorded included:
o Jump height (cm)
o Flight time (ms)
o Take-off velocity (m/s)
o Estimated force (N)
o Power output (W)

All trials were recorded and analyzed by the same evaluator to ensure standardization and minimize inter-rater variability.

### 2.4 Statistical analysis

All statistical analyses were conducted using IBM SPSS Statistics for Windows, Version 26.0 (IBM Corp., Armonk, NY, United States of America). Prior to inferential analysis, the assumption of normality was evaluated using the Shapiro–Wilk test, due to its robustness in small sample sizes (n < 50). All performance variables were confirmed to follow a normal distribution (p > 0.05), thus allowing for the application of parametric statistical tests.

To assess differences in performance outcomes (vertical jump height and 30-m sprint time) across the three warm-up conditions (Control, Static Stretching, and RAMP), a one-way repeated-measures analysis of variance (ANOVA) was employed. This method was selected to control for within-subject variability and to maximize statistical power by accounting for inter-individual differences.

When Mauchly’s test indicated a violation of the sphericity assumption, Greenhouse–Geisser corrections were applied. In the case of statistically significant main effects (p < 0.05), *post hoc* pairwise comparisons were conducted using the Bonferroni correction to adjust for multiple comparisons and control the family-wise error rate.

Effect sizes were also calculated and reported as partial eta squared (η^2^_p_) to provide an indication of the practical relevance of any observed effects. All results are presented as mean ± standard deviation (SD) unless otherwise stated. The level of statistical significance was set at α = 0.05 for all analyses.

## 3 Results

The sociodemographic characteristics of the participants are presented in Table 3.

**TABLE 3:** Sociodemographic characteristics of the participants (n = 14).

Variable	Min	Max	Mean ± SD
Age (years)	20.00	23.00	21.86 ± 0.86
Height (cm)	177.00	185.00	180.42 ± 2.59
Weight (kg)	66.00	92.00	73.43 ± 7.90
BMI (kg/m^2^)	20.00	28.00	22.07 ± 2.76

Min, minimum; Max, maximum; SD, standard deviation; BMI, body mass index; cm, santimeter; kg, kilogram.

### 3.1 Vertical jump performance

**FIGURE 1:**
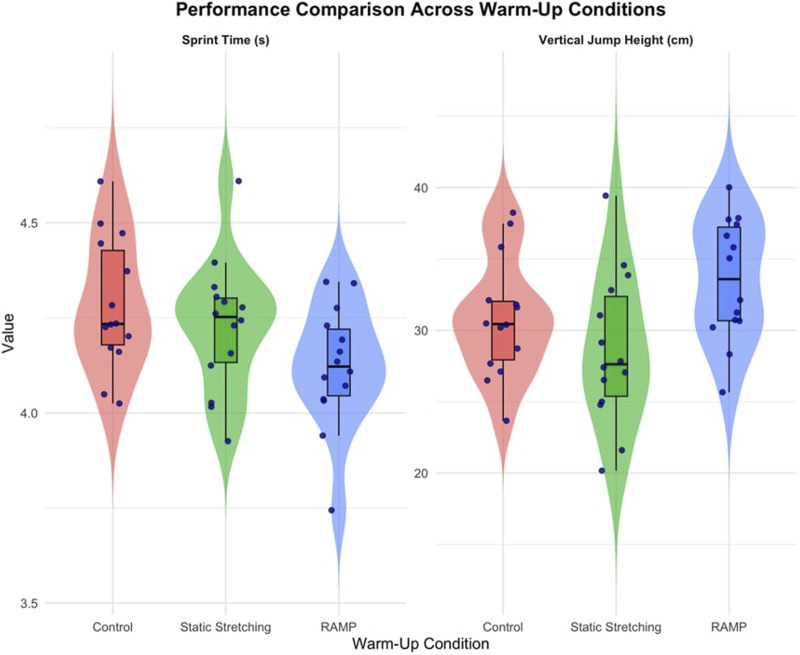
Box plots showing the distribution of vertical jump height and sprint time across the three warm-up conditions.

A statistically significant main effect of warm-up condition on vertical jump height was observed (*F* (2, 26) = 10.818, *p* < 0.001, ηp^2^ = 0.454), indicating a large effect size. Post hoc Bonferroni analyses revealed that the RAMP condition (32.49 ± 6.00 cm) significantly outperformed both the static stretching condition (30.26 ± 5.13 cm, *p* = 0.037) and the control condition (29.85 ± 4.89 cm, *p* = 0.048). No statistically significant difference was observed between the static stretching and control conditions (Table 4; Figure 1).

**TABLE 4:** Comparison of vertical jump and sprint performance means under different warm-up conditions (n = 14).

Parameter	Control (mean ± SD)	Static stretching (mean ± SD)	RAMP (mean ± SD)	F	p-value	ηp^2^
Jump Height (cm)	29.85 ± 4.89	30.26 ± 5.13	32.49 ± 6.00	10.818	<0.001*	0.454
Sprint Time (s)	4.24 ± 0.17	4.22 ± 0.19	4.16 ± 0.18	5.723	0.009 *	0.306

SD, standard deviation; ηp^2^, denotes the effect size (partial eta squared), *p < 0.05. p < 0.05 was considered statistically significant. Results with p=0.009 are denoted as highly significant, and p < 0.001 as extremely significant.

### 3.2 Sprint performance

Sprint times also showed significant variation across conditions (*F* (2, 26) = 5.723, *p* = 0.009, ηp^2^ = 0.306). Bonferroni-adjusted pairwise comparisons demonstrated that the RAMP protocol (4.16 ± 0.18 s) resulted in significantly faster sprint times than both static stretching (4.22 ± 0.19 s, *p* = 0.000) and the control condition (4.24 ± 0.17 s, *p* = 0.022). No statistically significant difference was found between the static stretching and control conditions (Table 4; Figure 1).

## 4 Discussion

The present study aimed to investigate the acute effects of the RAMP warm-up protocol—compared with static stretching and no warm-up—on vertical jump and sprint performance in young male soccer players. The main findings of the study indicate that the RAMP protocol produced significantly better outcomes in both sprint and vertical jump performance compared with static stretching and no warm-up conditions. These improvements are aligned with earlier findings that emphasize the benefits of dynamic, sport-specific warm-ups in elevating neuromuscular activation and optimizing power output (McMillian et al., 2006; Jeffreys, 2019). The RAMP protocol, by systematically increasing body temperature and activating key muscle groups through dynamic drills, likely facilitates enhanced recruitment of type II (fast-twitch) muscle fibers, which are primarily responsible for explosive efforts such as sprinting and jumping. Additionally, the warm-up may improve the viscoelastic properties of the muscle–tendon unit by raising intramuscular temperature and reducing passive stiffness, which enhances stretch-shortening cycle efficiency (Bishop, 2003; Fradkin et al., 2010). However, because our study did not measure physiological variables (e.g., core temperature, electromyographic activity) directly, the proposed mechanisms remain theoretical, which is a notable limitation.

In addition to performance improvements, the multi-component structure of the RAMP protocol has been associated with enhanced joint mobility, neuromuscular coordination, and potentially lower injury risk, particularly when consistently applied before high-intensity exercise (Afonso et al., 2023; Brooks and Cressey, 2013). The activation and mobilization phases—featuring dynamic drills such as lateral shuffles, hurdle walks, and sport-specific movement patterns—are designed to enhance joint range of motion and proprioceptive feedback (Brooks and Cressey, 2013; Skopal et al., 2024). The activation and mobilization phases—featuring dynamic drills such as lateral shuffles, hurdle walks, and sport-specific movement patterns—are designed to enhance joint range of motion and proprioceptive feedback. The ‘Activate’ phase of the RAMP protocol typically incorporates dynamic stretching activities—such as lunges, high knees, and leg swings—to engage key muscle groups and elevate neuromuscular readiness. Similar studies, such as Eken et al. (2022) in judo athletes and Cieśluk et al. (2024) in volleyball players, have shown that dynamic warm-up protocols, including RAMP phases, lead to improvements in neuromuscular function, agility, and even cognitive measures such as reaction time.

These benefits are likely driven by acute physiological adaptations triggered during warm-up routines. Increased muscle and core temperature enhances nerve conduction velocity, reduces musculotendinous stiffness, and improves oxygen delivery and metabolic enzyme activity—all of which contribute to more effective muscle fiber recruitment and increased power output during explosive tasks (Fradkin et al., 2010; McCrary et al., 2015; Barbosa et al., 2025a). Such mechanisms are consistent with recent calls for contemporary warm-up models that emphasize progression, individualization, and task specificity (Barbosa et al., 2025b). These findings suggest that the neuromuscular and biomechanical benefits of a RAMP-based warm-up may translate into greater overall readiness for performance. Nevertheless, our inability to directly assess changes in physiological variables such as core temperature or electromyographic activity limits the degree to which these mechanisms can be confirmed in the current study.

Furthermore, the significant improvement in performance following the RAMP protocol underscores its potential practical application in various sporting contexts. The integration of dynamic exercises that replicate in-game movements may enhance acute performance while promoting long-term adaptations in strength, agility, and coordination when applied systematically in training environments (Kukić et al., 2024; Skopal et al., 2024). This notion is supported by the work of Kukić et al. (2024), who observed meaningful improvements in change-of-direction speed over an extended intervention period.

Several limitations should be noted. First, the relatively small sample size reduces statistical power and limits generalizability. Second, the study focused exclusively on male soccer players, excluding other age groups, sexes, and sports. Third, only acute effects were measured, providing no information on long-term adaptations. Finally, physiological mechanisms underlying performance gains—such as core temperature or EMG activity—were not directly assessed. Future studies should aim to address these gaps by including larger and more diverse samples, integrating physiological monitoring, and evaluating chronic effects of warm-up strategies.

This study contributes to the growing body of literature validating structured warm-up protocols. By isolating the acute impact of the RAMP method in a real-world athletic population, the findings underscore its practical utility in boosting short-term performance. Coaches and sports scientists may benefit from integrating RAMP into standard practice as a low-cost, evidence-based strategy to optimize readiness and potentially reduce injury risk, especially in youth and amateur settings.

## 5 Conclusion

This study demonstrates that the RAMP warm-up protocol provides significant acute benefits for sprint and vertical jump performance in youth soccer players. Compared to static stretching and no warm-up, the RAMP protocol proved more effective in enhancing explosive power and neuromuscular readiness. These results align with existing literature and support the integration of RAMP as an evidence-based, structured warm-up method in athletic training environments. Coaches and practitioners are encouraged to adopt the RAMP framework to optimize short-term performance outcomes in competitive settings.

## Data Availability

The raw data supporting the conclusions of this article will be made available by the authors, without undue reservation.
